# Radiomics score predicts acute respiratory distress syndrome based on the initial CT scan after trauma

**DOI:** 10.1007/s00330-020-07635-6

**Published:** 2021-03-17

**Authors:** Sebastian Röhrich, Johannes Hofmanninger, Lukas Negrin, Georg Langs, Helmut Prosch

**Affiliations:** 1 Department of Biomedical Imaging and Image-guided Therapy, Medical University of Vienna, Vienna, Austria; 2 Computational Imaging Research Lab, Department of Biomedical Imaging and Image-Guided Therapy, Medical University of Vienna, Waehringer Guertel 18-20, A-1090 Vienna, Austria; 3 Department of Orthopedics and Trauma-Surgery, Medical University of Vienna, Vienna, Austria

**Keywords:** Thoracic injuries, Acute respiratory distress syndrome, Polytrauma, Multidetector computed tomography, Radiomics

## Abstract

**Objectives:**

Acute respiratory distress syndrome (ARDS) constitutes a major factor determining the clinical outcome in polytraumatized patients. Early prediction of ARDS is crucial for timely supportive therapy to reduce morbidity and mortality. The objective of this study was to develop and test a machine learning–based method for the early prediction of ARDS derived from the first computed tomography scan of polytraumatized patients after admission to the hospital.

**Materials and methods:**

One hundred twenty-three patients (86 male and 37 female, age 41.2 ± 16.4) with an injury severity score (ISS) of 16 or higher (31.9 ± 10.9) were prospectively included and received a CT scan within 1 h after the accident. The lungs, including air pockets and pleural effusions, were automatically segmented using a deep learning–based algorithm. Subsequently, we extracted radiomics features from within the lung and trained an ensemble of gradient boosted trees (GBT) to predict future ARDS.

**Results:**

Cross-validated ARDS prediction resulted in an area under the curve (AUC) of 0.79 for the radiomics score compared to 0.66 for ISS, and 0.68 for the abbreviated injury score of the thorax (AIS-thorax). Prediction using the radiomics score yielded an f1-score of 0.70 compared to 0.53 for ISS and 0.57 for AIS-thorax. The radiomics score achieved a sensitivity and specificity of 0.80 and 0.76.

**Conclusions:**

This study proposes a radiomics-based algorithm for the prediction of ARDS in polytraumatized patients at the time of admission to hospital with an accuracy that competes and surpasses conventional scores despite the heterogeneous, and therefore more realistic, scanning protocols.

**Key Points:**

*• Early prediction of acute respiratory distress syndrome in polytraumatized patients is possible, even when using heterogenous data.*

*• Radiomics-based prediction resulted in an area under the curve of 0.79 compared to 0.66 for the injury severity score, and 0.68 for the abbreviated injury score of the thorax.*

*• Highlighting the most relevant lung regions for prediction facilitates the understanding of machine learning–based prediction.*

## Introduction

Blunt thoracic trauma is a common injury mechanism in polytraumatized patients and of those, two-thirds may suffer from parenchymal lung injury [1]. The relevance of parenchymal injuries (lung contusion and laceration) lies in the increased risk of developing acute respiratory distress syndrome (ARDS) and, consequently, the resulting deterioration of clinical outcome [2–5]. Whereas lung contusion and the resulting damage of the alveolar epithelial cells are a direct factor in the genesis of ARDS [6–8], systemic inflammatory response mediators are important indirect pathomechanisms [9]. Predictive factors at admission to the hospital after blunt trauma for the development of ARDS comprise parenchymal lung injuries such as lung contusions, hypotension, necessity of blood transfusion, age higher than 65 years, and an injury severity score (ISS) higher than 25 [10, 11]. Depending on the specifics of the study cohort, incidence and mortality may range from 5.8 to 21.5 % and 12.2 to 24.9 % respectively, with a decrease of both incidence and mortality in the last years [12–17]. Furthermore, there is also a considerable socio-economic impact due to impaired cognitive and physical function after convalescence [18]. Currently, the accuracy of early prediction methods has an area under the curve (AUC) of 0.72 to 0.82 for trauma score [11, 19] and 0.67 to 0.75 for computed tomography (CT) volumetric methods [20, 21]. Early prediction of ARDS in polytraumatized patients may enable timely supportive therapy, such as adjusting ventilation for lung protection, restricting the administration of transfusions, or starting the administration of antibiotics [22, 23], and, thus, reduce complications or prevent the development of ARDS altogether.

CT of the chest is a widespread and essential tool in the primary diagnostic process of trauma patients after admission to the hospital [24]. The initial traumatic injury results in focal or diffuse alveolar hemorrhage [25] followed by a lung edema and interstitial alterations in the affected parenchyma [26]. While these imaging findings may prove difficult to interpret at the beginning, and only become more prominent in the ensuing 24 to 48 h [27], early information from the first CT scan would help to decide on the abovementioned pivotal therapeutic decisions. Furthermore, the patient’s condition may prevent a transport to the CT scanner and multiple exposures to ionizing radiation of the often young population of polytraumatized patients carry an increased risk of inducing oncological disease in survivors. Therefore, novel methods to raise as much information as possible from the first scan after admission to the hospital are crucial.

The European Society of Radiology endorses the extraction of quantitative biomarkers from medical images that inform on disease detection, characterization, monitoring, and assessment of response to treatment [28]. One method to extract information from images that is not entirely accessible for the human eye is radiomics [29], for which an increasing number of applications emerge in non-oncologic chest CT [30].

It was the aim of this study to develop and test a radiomics-based computational method for the early prediction of ARDS based on the initial CT scan in a cohort of prospectively included patients with chest trauma.

## Methods

### Data collection and study population

The imaging and clinical data acquired for this study were collected in the framework of a prospective study which aimed to evaluate manual volumetry of parenchymal lung injury in initial and follow-up CT scans to predict ARDS in polytraumatized patients [31]. In total, 123 of the previously reported patients were evaluated. The previous study reported on the usefulness of a follow-up chest CT scan for manual volumetry of lung parenchymal injuries compared to the initial scan at admission whereas the present study evaluated the possibility of machine learning–based prediction of ARDS in the initial scan.

The patients were included over a timeframe of 4 years. Inclusion criteria were 18 years or older, ISS of 16 or higher, direct transport to the study hospital (level I trauma center) with a CT scan within 1 h after the trauma, and admission to the intensive care unit. Informed consent was gained from the patient or legal representative. Exclusion criteria were death within 48 h (this was a methodological necessity of the original publication [31]), burning injury, oncological disease, and chronic inflammatory lung disease. The Berlin Definition [7] was used to define ARDS based on oxygenation, chest imaging, timing, and origin of edema. The CT-independent methods of establishing ARDS were partial pressure of arterial oxygen (PaO_2_) ≤ 300 mm Hg with positive end-expiratory pressure (PEEP) or continuous positive airway pressure ≥ 5 cm H_2_O, respiratory failure not fully explained by cardiac failure or fluid overload, and a development within 1 week of the trauma.

One hundred sixteen patients received intravenously administered contrast agent; for 7 patients, the administration of contrast agent was not possible. In patients who were hemodynamically unstable when reaching the emergency room (*n* = 6), we performed an arterial phase with a field of view ranging from the skull base to the proximal femur (bolus tracking technique at the aortic arch with a threshold of 100 Hounsfield units (HU) and 10-s delay; 120 ml in total with a flow of 4.5 ml/s for 31 s followed by 3.3 ml/s for 18 s), followed by a venous phase over the abdomen. All other patients received a venous phase over the chest and abdomen (120 ml in total with a flow of 3 ml/s over 55 s).

### Machine learning algorithm for ARDS prediction

**Fig. 1:**
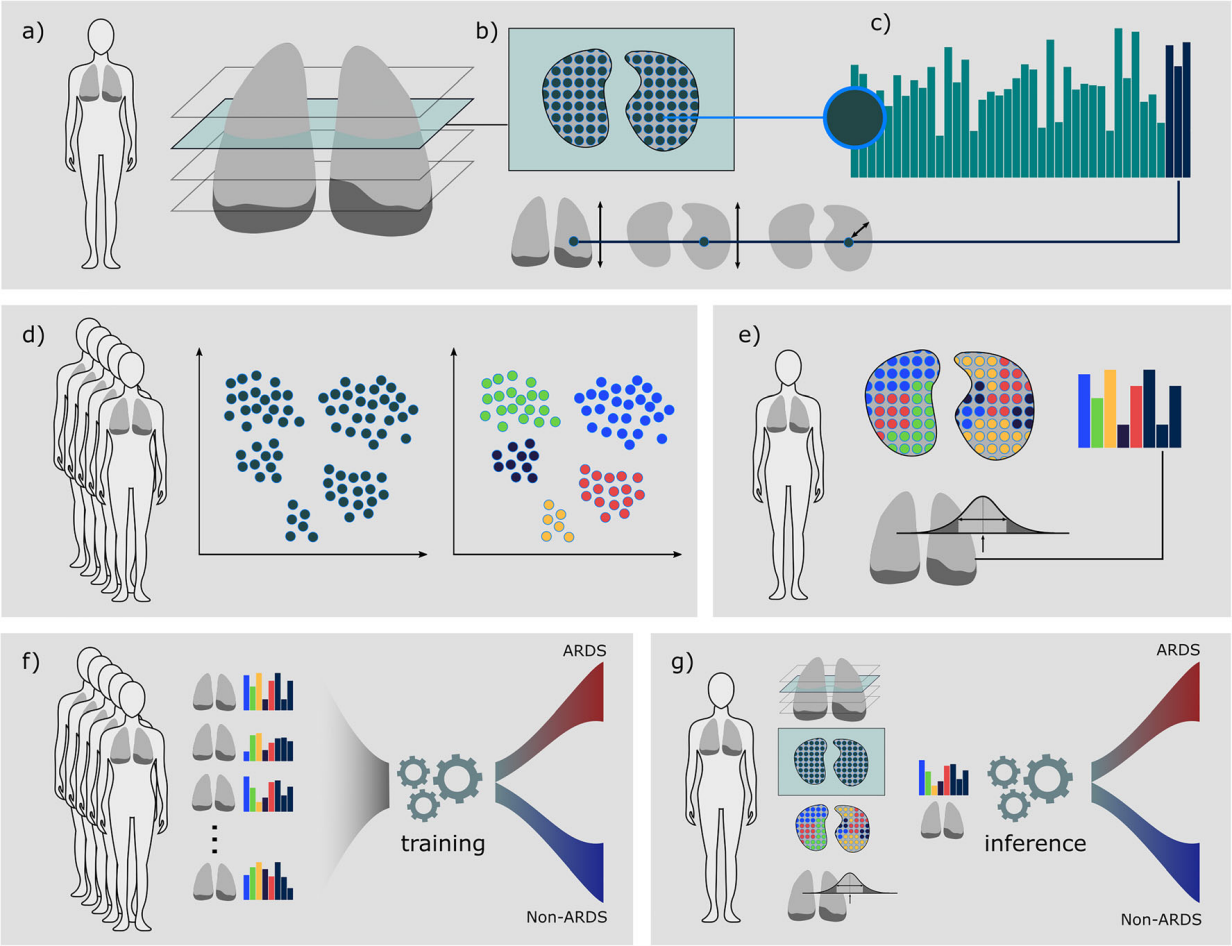
Overview—from CT scan to ARDS prediction: **a** We performed a machine learning–based segmentation of the lung, including effusion and air pockets. **b** From within the lung-mask, we extracted 2D radiomics features in a grid pattern over a kernel. In addition, we calculated the reference locations (anterior-posterior, inferior-superior, and the distance transform) to retrieve localized spatio-visual feature vectors for each location as illustrated in **c**. **d** A spatio-visual vocabulary sampled from feature vectors of the full dataset was learned. **e** After the vocabulary had been learned, a single patient is represented by his vocabulary histogram and statistical features calculated on the HU histogram of the full lung. **f** We trained a GBT ensemble on a training set of feature representations to distinguish cases that will develop ARDS in the future and cases that will not. **g** After training, prediction for a novel case is performed fully automated, from raw DICOM images, lung segmentation, and feature extraction to ARDS risk score. ARDS, acute respiratory distress syndrome; GBT, gradient boosted tree

Figure 1 gives an overview of the machine learning algorithm for the prediction of ARDS.

First, we segmented the lung with a convolutional neural network (CNN) approach to automatically delineate the lung including pleural effusions and air pockets (pneumothorax) [32]. Visual inspection by a radiologist showed a good fit of the segmentations to the thoracic cavity, excluding the mediastinum.

After lung segmentation in all cases, we learned a visual vocabulary to represent the appearance variability in the study cohort. Specifically, we extracted 83 2D radiomics features on a grid from axial slices throughout the lung with 1-cm distance between the locations and within a kernel diameter of 2.4 cm.

For the extraction of radiomics data, we have relied on pyradiomics, an open-source python package for the extraction of radiomics features [28]. Note that the pyradiomics libraries are research tools and not approved for clinical use.

We calculated first-order statistics and features on gray level co-occurrence, gray level run length, gray level size zone, and gray level dependence matrices. To ensure comparable radiomics feature across scans with variable voxel sizes, the image volumes were resampled to a voxel resolution of 0.8 × 0.8 × 2 mm [33]; voxel intensities were clipped at ‑ 990 and 200 HU. The extraction of texture features relies on the reduction of image intensities to a reduced number of discrete values. We quantized intensities into 40 bins (30 HU bin-width) for the calculation of the texture matrices. In addition to visual features, we calculated reference locations for each feature vector with three spatial coordinates (1) anterior-posterior, (2) inferior-superior, and the (3) distance to the lung border. Radiomics feature vectors together with the reference coordinate randomly sampled from all cases (*N* ≈ 24600 spatio-visual vectors) were used to learn a spatio-visual vocabulary, i.e., the feature vectors were clustered into 20 classes using k-means, each class describing a visual pattern.

Subsequently, we calculated the vocabulary occurrence feature vectors for each single case. That is, the proportions of each of the vocabulary classes in a case form a quantized radiomics (QR) feature vector capturing the composition of lung appearance patterns. In addition to these 20 values, we calculated 10 statistical features on all HU values within the lung: volume, mean, median, mean-average-deviation, variance, energy, % of HU ≥ 300, ≥ 200, ≥ 100, and > 0.

Finally, we used these 30 lung features for machine learning–based prediction of whether the patient would develop ARDS or not by training a Gradient Boosted Tree (GBT) ensemble classifier [34] with 1k decision trees as provided by the scikit-learn library [35]. After training, ARDS risk prediction is performed fully automated, including lung segmentation and feature extraction without any human interaction required.

To estimate the predictive capabilities of the machine learning model on unseen data, we performed stratified k-fold cross-validation, a resampling procedure used to evaluate models on a limited data sample. In our study, 40 subsets of the original cases were obtained by randomization, and then every subset was validated against the remaining 39 subsets as training data, repeating this procedure 40 folds. Each fold in our study was composed of 39 ARDS cases, 81 (‑ 1) non-ARDS in the training and one ARDS, 2 (+ 1) non-ARDS cases in the test set. During each fold, the training data were used to optimize the model parameters while the test data were only used to report the prediction score.

The prediction performance was analyzed with a receiver operating characteristic (ROC) curve and additional prediction metrics (sensitivity, specificity, precision, and f1 score) at the cutoffs yielded by the highest Youden index.

In addition to the supervised prediction experiments, we performed unsupervised cluster analysis based on the feature vectors. Statistically, a *χ*^2^ test was used to analyze the relationship between future ARDS status and the two main clusters formed by the radiomics feature expression. We report box plots and two-tailed *t* tests to compare the non-ARDS and ARDS groups with respect to their risk scores (radiomics risk score, AIS, and ISS).

We performed statistical association tests to investigate potential spurious relationships between factors that may influence image appearance and future ARDS status. Specifically, we tested for associations between future ARDS and contrast agent administration, scanner model, slice thickness, reconstruction kernel, and dose. Furthermore, we calculated the evaluation scores (AUC, sensitivity, specificity, precision, and f1 score) for subsets of the population with homogeneous image parameters less influenced by these potential biases.

### Statistical analysis

All statistical measures and tests were performed with the scipy statistics package (v 1.3.1) [36]. To test the associations between factors that may influence image appearance and future ARDS status, the Fisher exact tests have been performed for categorical variables (contrast agent administration, scanner model, slice thickness, and reconstruction kernel) and a two-tailed *t* test has been performed for dose (mAs).

## Results

In total, 123 patients with different blunt accident mechanisms (most common causes were pedestrian vs. vehicles (23.4%), fall from 3 m or higher (25.9%), and motor vehicle accidents (38.8%)) were included. Death occurred in five of the included patients (4%), of whom three died from ARDS, one from multi-organ failure of different etiology, and one from brain injury. Detailed demographic characteristics of the study population are shown in Table 1, an overview of technical specifications of the CT scans is shown in Table 2, and thoracic injury patterns are listed in Table 3.

**Table 1:** Characteristics of the study population

Patient characteristics	Mean ± Std; number (%)
Male/female	86/37
Age	41.2 ± 16.4
ISS	31.9 ± 10.9
AIS-thorax 0 AIS-thorax 1 AIS-thorax 2 AIS-thorax 3 AIS-thorax 4 AIS-thorax 5	11 3 13 38 33 25
Patients arriving intubated at the trauma center Patients with a chest tube insertion at the site of injury Patients with a chest tube insertion at the hospital Patients needing extracorporeal membrane oxygenation (ECMO) Patients developing acute respiratory distress syndrome (ARDS)	68 (55.3%) 15 (12.2%) 41 (33.3%) 3 (2.4%) 40 (32.5%)

*ISS* injury severity score, *AIS-thorax* abbreviated injury score of the thorax

**Table 2:** Technical specifications of the CT scans

	Value	Number of cases
Slice thickness (mm)	1.5	38
	2.0	37
	3.0	41
	5.0	7
In-plane pixel spacing (mm)	0.56 × 0.56 to 0.98 × 0.98	123
Tube voltage (kV)	120	122
	140	1
Exposure (mAs)	39 to 467	123
Scanner	Sensation 16	3
	Sensation 4	11
	Sensation Cardiac 64	6
	Sensation Open	102
	SOMATOM Definition	1
Convolution kernel	B60s	3
	B60f	5
	B70s	1
	B70f	114
Contrast phase	Non-contrast	7
	Arterial	6
	Venous	110

*mm* millimeter, *kV* kilovolt, *mAs* milliampere second

**Table 3:** Thoracic injury patterns in the study population

Pathology	Location	Frequency per side	Frequency in total
Lung contusion	Unilateral	51 (41.5%)	82 (66.7%)
	Bilateral	31 (25.2%)	
Lung laceration	Unilateral	21 (17.1%)	22 (17.9%)
	Bilateral	1 (0.8%)	
Pneumothorax	Unilateral	43 (35.0%)	49 (39.8%)
	Bilateral	6 (4.9%)	
Hematothorax	Unilateral	7 (5.7%)	9 (7.3%)
	Bilateral	2 (1.6%)	
Hematopneumothorax	Unilateral	7 (5.7%)	8 (6.5%)
	Bilateral	1 (0.8%)	
Rib fractures	1 rib	16 (13.0%)	87 (70.7%)
	2 ribs	7 (5.7%)	
	≥ 3 ribs	64 (52.0%)	
Flail chest			21 (17.1%)
Sternum fracture			25 (20.3%)
Thoracic spine fracture			37 (30.1%)
Extensive surgical emphysema			21 (17.1%)
Aortic dissection			7 (5.7%)
Diaphragmatic rupture			2 (1.6%)
Pneumomediastinum			6 (4.9%)

One hundred one (77.7%) of the 123 patients had an abbreviated injury scale (AIS) of the thorax of 3 or higher, indicating a severe thoracic injury [37], of which 93 (71.5% of 101) had a parenchymal lung injury.

**Fig. 2:**
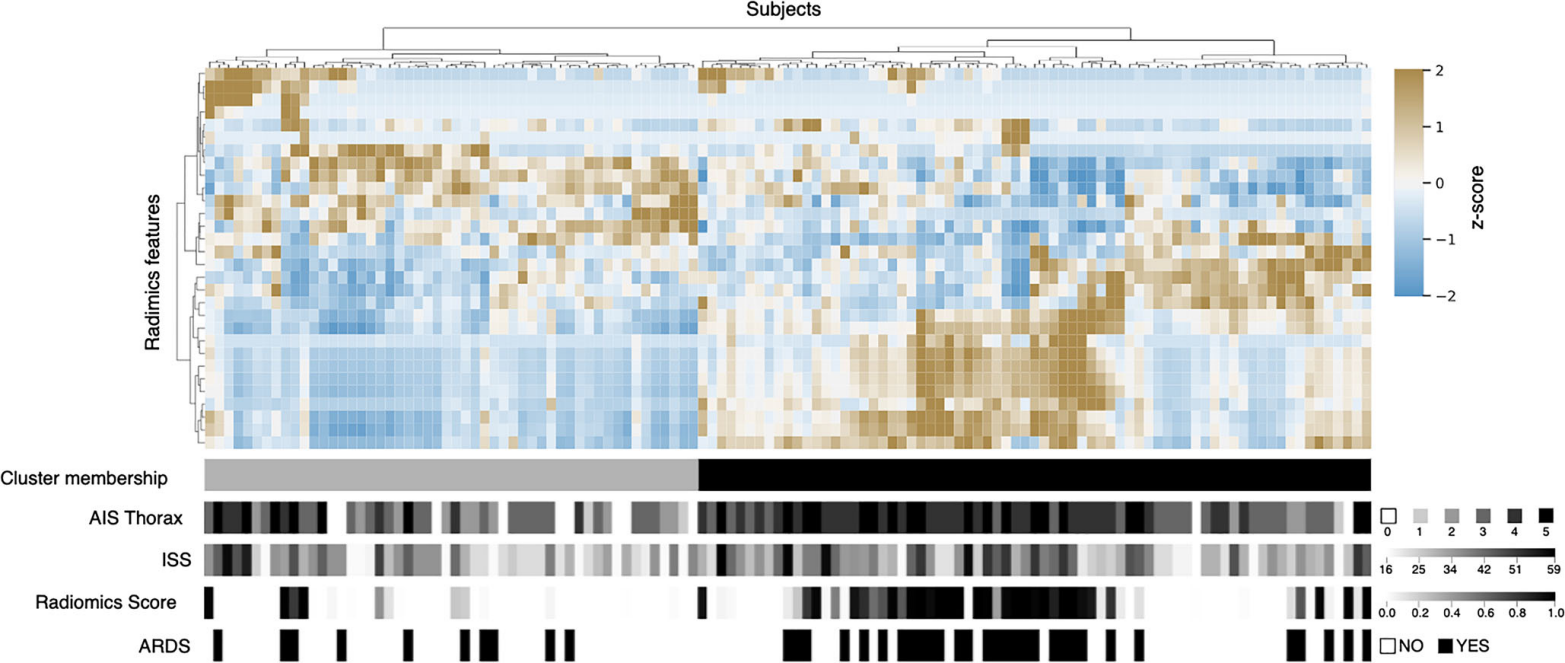
Feature heatmap and unsupervised clustering: **C**lustered feature expressions and their association with injury severity scores, the machine learning–based radiomics risk score, and the future ARDS status. Each column represents one patient sorted after agglomerative clustering. A *χ*^2^ test reveals a significant relationship (*p* = 0.012) between the two main clusters formed by the radiomics feature expressions and the future ARDS status

To investigate feature expression patterns, we performed unsupervised clustering revealing groups of patients with similar feature patterns (Fig. 2). We compared the two main clusters of patients with their future ARDS status and found a significant association (*p* = 0.012, *χ*^2^ test).

**Fig. 3:**
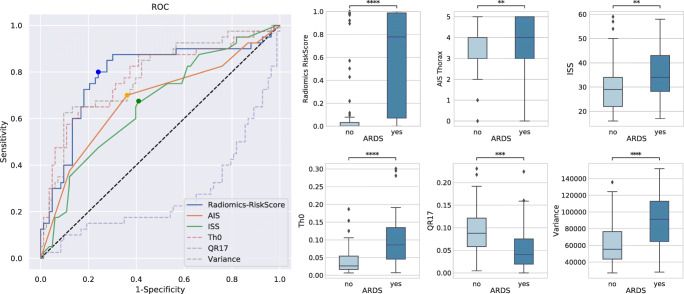
Quantitative results: This receiver operating characteristic (ROC) curve shows the superior performance of radiomics-based prediction of acute respiratory distress syndrome (ARDS) compared to conventional trauma scores. In addition, the ROC curves for the three most relevant features as reported by the GBT ensemble are shown and the scores and feature expressions are compared between ARDS and non-ARDS cases via boxplots and *t* tests. **: 1.00e-03 *< p* < = 1.00e-02; ***: 1.00e-04 < *p* < = 1.00e-03; ****: *p* < = 1.00e-04. ISS, injury severity score; AIS, abbreviated injury scale (for thorax)

**Fig. 4:**
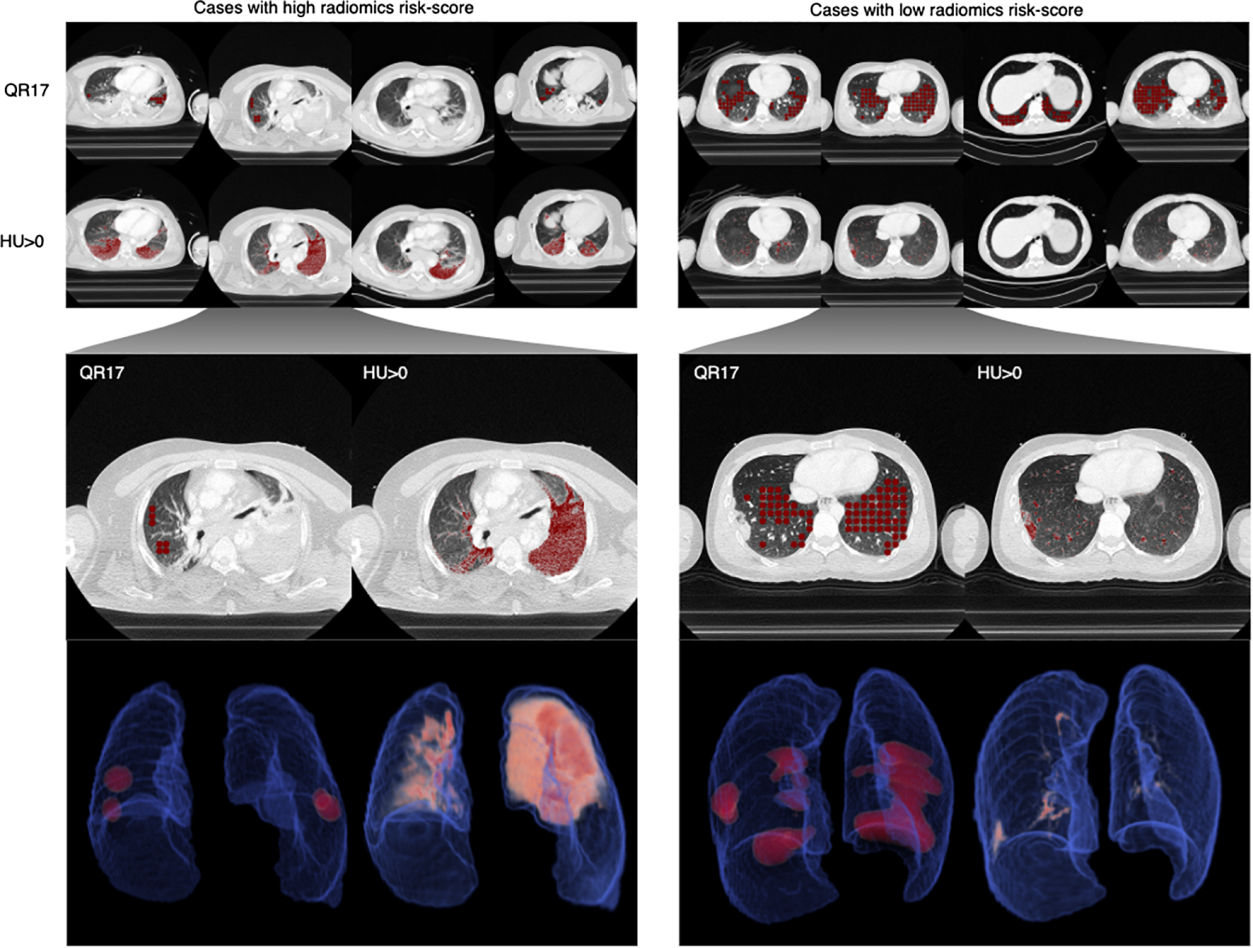
Visualization of predictive features: Here, the two most relevant visual features for ARDS prediction (QR17 and HU > 0) as reported by the GBT ensemble are visualized. On the left side, the four cases which received the highest risk score are shown and on the right side the four cases which received the lowest risk score. In addition, a 3D visualization of the locations of the features is shown in two representative cases

Forty-fold cross-validation of ARDS prediction resulted in an AUC of 0.79 for the GBT-based radiomics score compared to 0.66 for ISS, and 0.68 for the AIS-thorax score (Fig. 3). Additional prediction metrics such as sensitivity, specificity, precision, and f1 score can be found in Table 4 (cutoffs at the highest Youden index are plotted in Fig. 3). Prediction using the radiomics score yielded an f1 score of 0.70 compared to 0.53 for ISS and 0.57 for AIS-thorax. The radiomics score achieved a sensitivity/specificity of 0.80/0.76. The features with the highest relative importance for classification yielded by the GBT ensemble during training were quantized radiomics feature 17 (QR17), HU > 0, and variance, with HU > 0 and variance showing positive correlation with future ARDS and QR17 showing a negative correlation. In Fig. 4, we illustrate the most relevant features HU > 0 and QR17 in cases with a high radiomics risk score for ARDS and cases with a low risk score.

**Table 4:** Results for the prediction of acute respiratory distress syndrome

Prediction based on	Sensitivity	Specificity	Precision	f1 score	AUC
ISS	0.68	0.59	0.44	0.53	0.66
AIS	0.70	0.64	0.48	0.57	0.68
Radiomics score (whole dataset)	0.80	0.76	0.62	0.70	0.79
Radiomics score (subgroup 1)	0.81	0.79	0.57	0.67	0.79
Radiomics score (subgroup 2)	0.81	0.71	0.59	0.69	0.75
Rib fractures (yes/no)	0.75	0.31	0.34	0.47	

*ISS* injury severity score, *AIS* abbreviated injury scale (for thorax), *AUC* area under the curve, *subgroup 1* only cases scanned during a venous phase, scanner model S5, and kernel B70f, *subgroup 2* only cases with 2-mm or 3-mm slice thickness

Table 5 lists the relationships between factors that may influence image appearance and future ARDS status. The results indicate that the correlation between the scanner model and slice thickness with future ARDS status may not likely have occurred randomly. To assess the results less influenced by these potential biases, we performed an evaluation of the prediction model on two subgroups with homogeneous parameters: (subgroup 1) only cases scanned during the venous phase, scanner model S5 (see Table 5) and a high-frequency reconstruction algorithm (kernel B70f) and (subgroup 2) only cases with either slice thickness 2 mm or 3 mm. The results show that the model remains predictive on a level nearly the same as the whole dataset, indicating a negligible influence of the tested factors with an f1 score of 0.70 for the full population, 0.67 for subgroup 1, and 0.69 for subgroup 2 (also see Table 4).

**Table 5:** Assessment of potential technical biases

	Contrast			Scanner model					Slice thickness (mm)				Kernel				Dose (mAs)	
	n	a	v	Sc1	Sc2	Sc3	Sc4	Sc5	1.5	2	3	5	B60f	B60s	B70s	B70f	Mean	std
No ARDS	4	3	76	0	0	6	3	74	31	22	29	1	2	1	1	79	189	57
future ARDS	3	3	34	1	3	5	3	28	7	15	12	6	3	2	0	35	188	65
*p* value	0.681	0.389	0.348	0.325	0.033	0.335	0.389	0.011	0.036	0.217	0.685	0.005	0.328	0.247	0.149	1.000	0.92	
Test	Fisher			Fisher					Fisher				Fisher				*t* test	

*ARDS* acute respiratory distress syndrome, *n* no contrast, *a* arterial phase; *v* venous phase, *Sc1* SOMATOM Definition, *Sc2* Sensation 16, *Sc3* Sensation 4, *Sc4* Sensation Cardiac 64, *Sc5* Sensation Open, *mAs* milliampere second, *std* standard deviation, *Fisher* Fisher exact test

## Discussion

The early recognition of a developing ARDS in the hours and days after trauma is critical for timely initiation of adequate treatment (such as adjusting ventilation for lung protection, preventing aspiration, restricting the administration of transfusions, or administration of antibiotics) which may decrease the incidence rate of full ARDS [22, 23]. In the present study, we could demonstrate that a fully automated machine learning– and radiomics-based approach can identify polytraumatized patients with an increased risk of developing ARDS with higher accuracy than established scores by utilizing the first CT scan at the time of admission.

Currently, trauma scores such as the ISS or thoracic trauma severity (TTS) score can be used to estimate the risk of delayed ARDS after trauma [11, 19, 38]. However, the ISS is a combined score of different body regions and, thus, is prone to error due to the lack of weighting of different regions. With the proposed radiomics-based approach, a more specific representation of the lung’s injuries could be achieved. Indeed, the accuracies for the prediction of ARDS are at a similar level or higher than what was achieved in several studies using trauma scores (this study: AUC of 0.79, ISS: AUC of 0.72 [11], TTS: AUC of 0.82 [19]). However, another study could show better results for the ISS with an AUC of 0.88 [12]. Additionally, the ISS score in our study showed markedly worse performance (AUC of 0.66) than the ISS scores in all of the aforementioned studies. A possible reason can be found in the different inclusion criteria: whereas the previous study included all patients regardless of ISS score, our study only included patients with an ISS score of 16 or higher. In a cohort that includes low ISS scores, the performance in predicting ARDS will be higher as low ISS scores will lead to more true-negative cases [12]. In our study, the accuracy is reduced by patients that are more heavily injured, but not injured enough to easily predict the occurrence of ARDS. Thus, a radiomics-based approach may help in further stratifying a more heavily injured patient cohort for which the ISS score has reduced predictive capabilities.

One reason for this may be the importance of direct lung injury as an independent risk factor for the development of ARDS, in particular forms of parenchymal lung injuries such as lung contusions or lacerations [11]. Parenchymal lung injuries cause localized and generalized inflammatory reactions that may lead to ARDS [39, 40]. Consequently, both studies involving CT and ultrasound have found a positive correlation of the extent of lung contusions with the development of ARDS [20, 21, 31, 41, 42].

However, manual volumetry of parenchymal lung injuries is a very time-consuming task and not fit for clinical application, especially in the time-critical setting of trauma care. Finally, disregarding other pulmonary or pleural pathologies after trauma (e.g., hemothorax) could reduce the accuracy for predicting ARDS. A study investigating chest injury patterns after blunt trauma has shown pleural collections to be present in ~ 30% of all cases and in 83% of fatal cases [43]. A hemothorax is important for the prognosis of morbidity or mortality after trauma in two ways: on the one hand, by indicating bleeding from lung laceration or due to vessel injury and, on the other hand, by constituting a risk factor for the development of restrictive and infectious pleural processes, and consequently, respiratory failure [44]. Considering the relevance of a hemothorax, we included pleural effusions as part of the lung segmentations. The visualization of predictive features (see Fig. 4) suggests a high relevance for increased densities in the posterior pleural space, attributable to hemothorax, and for increased lung parenchymal densities, such as in parenchymal lung injuries.

Increased lung densities can represent one or several underlying pathologies (i.e., parenchymal lung injuries, edema, bleeding from another lung region, atelectasis). A clear statement about the densities’ etiology from images alone is often not possible for radiologists. Consequently, a study that applied a semiautomatic method based on thresholds of elevated lung density resulted in a better prediction of ARDS occurrence than human readers [45]. The advantage of a radiomics-based approach is the possibility of providing additional information beyond what human vision is capable of by not only assessing density thresholds, but by including complex statistical relationships of voxels [29]. Feature importance analysis showed that a generic threshold (HU > 0) has high relevance and a positive association with future ARDS status. However, one of the fine-grained appearance classes (QR17) showed high relevance and a negative association. Visual inspection indicates that this feature encodes a form of inconspicuous parenchyma texture.

In trauma, both pulmonary and extrapulmonary risk factors influence the course of ARDS development, and, with an accuracy of 71%, it is possible to assign an extrapulmonary origin to a typical, more symmetric pattern of ARDS in chest CT [46]. It would be interesting to assess the performance of a radiomics analysis to discriminate between ARDS of pulmonary and extrapulmonary origin. Regarding the prediction of ARDS based on lung alterations that occur during or immediately after the trauma, it seems unlikely that our proposed approach can reliably predict ARDS of extrapulmonary origin as CT images of lung parenchymal changes are the sole input of the method.

One known extrapulmonary predictor of ARDS is rib fracture [47]. Whereas our method focused on a radiomics-based approach to extract information from parenchymal and pleural pathologies, adding rib fractures as a categorical or ordinal variable might increase the accuracy of ARDS prediction. Automated rib fracture detection on a per case basis (i.e., are rib fractures present or not in a patient) already exists and could be considered an addition in further research [48]. Extrapulmonary predictors, such as cardiopulmonary or hematologic disease [47] or administration of fluids and transfusions [11], provide further relevant information. However, the clinical utility is hampered as in trauma patients it may be impossible to raise a comprehensive history or clinical parameters may only be available later on. Still, relevant information for the prediction of ARDS can be extracted from the electronic health record within the first 6 h after admission to the hospital by using a machine learning–based approach [49].

In addition to a risk estimation in an acute setting, a radiomics-based lung score could also be utilized for monitoring intensive care unit (ICU) patients. The complex course of disease progression and extensive data of ICU patients render the correct interpretation of a patient’s state a challenging task for clinicians. A recent study has shown the possibility of integrating various clinical parameters by a machine learning–based algorithm to predict complications (i.e., bleeding, renal failure, and mortality) of ICU patients at a level higher than conventional clinical risk models [50]. Such algorithms have the potential to take a pivotal supportive role in prediction, diagnosis, and monitoring of ICU patients and, furthermore, are expandable by adding other forms of data. Whereas a conventional model may struggle incorporating laboratory data and vital functions with medical imaging information, a machine learning model is able to include radiomics features as additional data.

We recognize several limitations of this study. We excluded patients with an ISS of 15 or lower; therefore, we can only assess the predictive capabilities of the proposed algorithm in a collective with major injuries and an increased risk of ARDS. However, the relatively low predictive value of ISS scores in this study’s dataset of polytraumatized patients compared to collectives that including less severe injuries (0.66 vs. 0.72 in [11]) indicates a more challenging setting. Furthermore, our cohort may be biased toward “late” ARDS (occurring > 48 h after trauma) compared to “early” ARDS (< 48 h) due to the exclusion of patients who died within 48 h.

To eliminate possible biases through heterogeneous scanner parameters, a completely standardized protocol on the same scanner would be preferential. In real clinical situations, particularly across different institutions, such preconditions are rarely met. Some of the scanner parameters that may influence radiomics features in this study are different flow rates and time after contrast agent administration [51], as well as reconstruction kernel, dose, slice thickness, and scanner type [52]. This is relevant because a machine learning model might learn to differentiate patients within the study population based on technical features, rather than the pathological features. In this study, 3 of 7 (43%) patients with a non-contrast CT, 3 of 6 (50%) patients with an arterial phase CT, and 34 of 110 (31%) patients with a venous phase CT developed ARDS. We tested for such potential biases, assessed the predictive performance in a more homogeneous subgroup, and came to the conclusion that the influence on the model’s predictive performance is negligible in our dataset (see the “Results” section, Tables 4 and 5). Interestingly, a correlation between a specific scanner and future ARDS occurrence may be due to the fact that more severely injured patients get transferred to a different scanner when the primary emergency room scanner is occupied, whereas less severely injured patients are kept on hold until the primary scanner is vacant again. Such organizational specifics should be kept in mind as a potential selection bias.

The features with the highest predictive capability were markedly increased densities in the posterior region of the thorax. While the timing of acquisition after injection of contrast agent may lead to a diffuse increase in HU of the lung, which theoretically might be learned by the algorithm, the visualization of relevant features suggests that the prediction of ARDS was based on regional abnormalities, rather than the systematic enhancement.

Furthermore, a standardization of inspiration depth is not possible for patients who experience severe chest pain or those who are intubated and with critical vital parameters, making respiration-based changes in lung density another potential source of noise. In the same manner, the elapsed time between trauma and CT scan may lead to alterations in lung densities as these may change rapidly depending on their etiology (e.g., bleeding, edema, atelectasis). In this study, all scans were conducted within one hour after the trauma.

Generally, a radiomics-based algorithm is agnostic towards predefined concepts of biology and pathology. Therefore, patterns and relationships discovered by such an approach are not directly attributable to common radiological patterns. However, they facilitate hypothesis generation for future studies through the discovery of novel patterns and their visual or statistical relationships.

In summary, we propose a radiomics-based algorithm for the prediction of ARDS in polytraumatized patients at the time of admission to hospital with an accuracy that competes and surpasses conventional scores despite the heterogeneous, and therefore more realistic, scanning protocols. Due to the generic nature of radiomics features, this algorithm may constitute a foundation for future, more complex models that integrate medical imaging information with clinical parameters. Furthermore, patterns identified as predictive signatures for ARDS may serve as a basis for hypotheses regarding underlying biological mechanisms.

## Abbreviations

AIS-thorax: Abbreviated injury score of the thorax
ARDS: Acute respiratory distress syndrome
AUC: Area under the curve
CNN: Convolutional neural network (CNN)
CT: Computed tomography
GBT: Gradient boosted trees
HU: Hounsfield units
ICU: Intensive care unit
ISS: Injury severity score
TTS: Thoracic trauma severity
