# Correction to: Radiomics score predicts acute respiratory distress syndrome based on the initial CT scan after trauma

**DOI:** 10.1007/s00330-021-07995-7

**Published:** 2021-05-04

**Authors:** Sebastian Röhrich, Johannes Hofmanninger, Lukas Negrin, Georg Langs, Helmut Prosch

**Affiliations:** 1 Department of Biomedical Imaging and Image-guided Therapy, Medical University of Vienna, Vienna, Austria; 2 Computational Imaging Research Lab, Department of Biomedical Imaging and Image-Guided Therapy, Medical University of Vienna, Waehringer Guertel 18-20, A-1090 Vienna, Austria; 3 Department of Orthopedics and Trauma-Surgery, Medical University of Vienna, Vienna, Austria

**Correction to: European Radiology.**

10.1007/s00330-020-07635-6

The original version of this article, published on 17 March 2021, unfortunately contained a mistake. The following correction has therefore been made in the original: The presentation of Fig. 3 was incorrect. The corrected figure is given below.

**Fig. 3:**
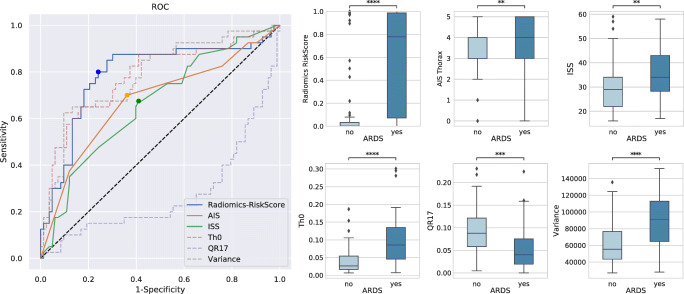
Quantitative results: This receiver operating characteristic (ROC) curve shows the superior performance of radiomics-based prediction of acute respiratory distress syndrome (ARDS) compared to conventional trauma scores. In addition, the ROC curves for the three most relevant features as reported by the GBT ensemble are shown and the scores and feature expressions are compared between ARDS and non-ARDS cases via boxplots and t tests. **: 1.00e-03 < *p* < = 1.00e-02; ***: 1.00e-04 < *p* < = 1.00e-03; ****: *p* < = 1.00e-04. ISS, injury severity score; AIS, abbreviated injury scale (for thorax)

